# Tumor-resident memory T cells as a biomarker of the response to cancer immunotherapy

**DOI:** 10.3389/fimmu.2023.1205984

**Published:** 2023-07-20

**Authors:** Isabelle Damei, Tatiana Trickovic, Fathia Mami-Chouaib, Stéphanie Corgnac

**Affiliations:** Institut National de la Santé et de la Recherche Médicale (INSERM) UMR 1186, Integrative Tumor Immunology and Immunotherapy, Gustave Roussy, Fac. de Médecine - Univ. Paris-Sud, Université Paris-Saclay, Villejuif, France

**Keywords:** tumor-resident memory T (TRM) cells, CD103 integrin, tumor microenvironment, cancer immunotherapy, biomarker

## Abstract

Tumor-infiltrating lymphocytes (TIL) often include a substantial subset of CD8^+^ tissue-resident memory T (T_RM_) cells enriched in tumor-specific T cells. These T_RM_ cells play a major role in antitumor immune response. They are identified on the basis of their expression of the CD103 (α_E_(CD103)β_7_) and/or CD49a (α_1_(CD49a)β_1_) integrins, and the C-type lectin CD69, which are involved in tissue residency. T_RM_ cells express several T-cell inhibitory receptors on their surface but they nevertheless react strongly to malignant cells, exerting a strong cytotoxic function, particularly in the context of blocking interactions of PD-1 with PD-L1 on target cells. These T_RM_ cells form stable conjugates with autologous tumor cells and interact with dendritic cells and other T cells within the tumor microenvironment to orchestrate an optimal *in situ* T-cell response. There is growing evidence to indicate that TGF-β is essential for the formation and maintenance of T_RM_ cells in the tumor, through the induction of CD103 expression on activated CD8^+^ T cells, and for the regulation of T_RM_ effector functions through bidirectional integrin signaling. CD8^+^ T_RM_ cells were initially described as a prognostic marker for survival in patients with various types of cancer, including ovarian, lung and breast cancers and melanoma. More recently, these tumor-resident CD8^+^ T cells have been shown to be a potent predictive biomarker of the response of cancer patients to immunotherapies, including therapeutic cancer vaccines and immune checkpoint blockade. In this review, we will highlight the major characteristics of tumor T_RM_ cell populations and the possibilities for their exploitation in the design of more effective immunotherapy strategies for cancer.

## 1 Introduction

The memory T-cell population includes central memory T (T_CM_) cells, which reside in lymphoid organs and can be reactivated by secondary infection with the same pathogen, and effector memory T (T_EM_) cells, with cytotoxic properties for CD8^+^ T lymphocytes, that patrol lymphoid and non-lymphoid peripheral tissues. More recently, a third subset of memory T cells, tissue-resident memory T (T_RM_) cells, was identified in peripheral tissues and intestine grafts (1, 2). In mice, this population of memory T cells was characterized in grafts, but not in peripheral blood, and seemed to be tissue-restricted (1, 2). Tissue transplant and parabiosis experiments have shown that the principal property of T_RM_ cells is their residence in peripheral tissues, with an inability to recirculate in the bloodstream (1–7). T_RM_ cells have been described in diverse human and mouse peripheral tissues, including the tissues of the intestine (2, 8), brain (9), skin (1, 10) and lung (11), in which they can confer rapid and effective immune responses to reinfections (12). T_RM_ cells are characterized principally by surface expression of the CD103 (α_E_(CD103)β_7_) and CD49a (VLA-1 or α_1_β_1_) integrins, and the C-type lectin CD69, although phenotype and integrin expression have been observed to differ between tissues (13, 14).

A subset of tumor-infiltrating lymphocytes (TIL) with several properties in common with T_RM_, including the expression of the CD103 integrin and CD69, has recently been identified in human tumors, including ovarian, lung and breast tumors, and melanoma (15–19). These CD103^+^CD8^+^ T cells accumulate in tumor regions, where they can interact with target cells to trigger their effector functions. An intra-epithelial location of CD103^+^CD8^+^ T cells was also observed in colorectal and bladder tumors with or without the expression of E-cadherin, the ligand of CD103, on cancer cells (20, 21). A high density of CD8^+^ T_RM_ cells within the tumor was found to be associated with better patient survival in multiple cancers, including melanoma, glioma, non-small cell lung carcinoma (NSCLC), and ovarian, bladder and breast cancers (15, 16, 18, 20–25). Abundant tumor infiltration with CD8^+^ T_RM_ cells is also associated with a stronger response of cancer patients to immunotherapies, including immune checkpoint blockade (ICB) and therapeutic cancer vaccines (17, 25, 26). In this review, we will summarize recent findings regarding the phenotypic and functional features of tumor T_RM_ cells and their role in antitumor immunity and response in cancer immunotherapies.

## 2 Characterization of T_RM_ cells in solid tumors

### 2.1 CD8^+^ T_RM_ cells

CD8^+^ T_RM_ cells can be identified on the basis of their surface expression of particular adhesion molecules and activation receptors. The receptors expressed on CD8^+^ T_RM_ cells include the CD69 lectin, which is known to bind to S1PR1, inhibiting its function and, thus, the egress of T cells from the tissue; it therefore contributes to the residency of these cells in the tissue (27). CD69 is not a specific marker of T_RM_; it is expressed on activated T-cells (28, 29) and on other types of memory T cells (30, 31). CD69 expression alone is not, therefore, sufficient to define T_RM_ cells. The expression of several integrins, particularly CD103 (α_E_β_7_) and CD49a (α_1_β_1_), has been reported to identify T_RM_ cells and to participate in the residency of these cells in peripheral tissues.

#### 2.1.1 The CD103 integrin, a key molecule for T_RM_ identification and functions

The CD103 integrin has two major functions on CD8^+^ T_RM_ cells: it is involved in the retention of T cells within epithelial tissues and it acts as a T-cell receptor (TCR) costimulatory molecule. Indeed, the binding of CD103 to its ligand, E-cadherin, contributes to the accumulation and maintenance of T_RM_ within tissues (9, 32–35). CD8^+^ T_RM_ cells can also express E-cadherin, which contributes to their own retention within tissues (36). CD103 neutralization decreases T-cell infiltration into tumors and aggravates tumor progression (37–40). CD103 also provides CD8^+^ T_RM_ cells with a TCR costimulation signal, and its interaction with E-cadherin strengthens adhesion to cancer cells and triggers the relocalization of cytotoxic granules to the immune synapse and target cell lysis (41–44). We have previously shown that the adhesion of CD103 to E-cadherin results in the binding of phosphorylated paxillin to the CD103 intracytoplasmic domain, initiating an outside-in signaling by CD103, thereby promoting CD8^+^ T_RM_ migration toward tumor cells and triggering T-cell effector functions (45).

#### 2.1.2 The CD49a integrin and other markers of CD8^+^ T_RM_ cells

Much less is known about the contribution of the CD49a integrin to T_RM_ cell functions. The binding of CD49a to collagen IV induces a relocalization of the integrin to focal adhesion points and increases T-cell anchorage to the extracellular matrix (ECM) (46). CD49a has been reported to promote T-cell retention in tissues, including those of the lungs (47), skin (14) and liver (48).

Another marker of CD8^+^ T_RM_ cells, CXCR6, has been reported to promote T-cell homing to the tissues and to regulate T_RM_ cell formation and maintenance (49, 50). Other chemokine receptors, such as CCR5 and CXCR3, are also expressed by tumor CD8^+^ T_RM_ cells and are probably involved in the residency of these cells in the inflammatory tumor microenvironment (16–18, 51, 52).

#### 2.1.3 Transcription factors involved in T_RM_ cell differention and persistence

T_RM_ cells undergo a specific differentiation program, distinct from that of memory T-cell lineage, such as by downregulating eomesodermin (EOMES) and T-cell factor 1 (TCF1) (53, 54). In mice, the expression of both Hobit and Blimp-1 transcription factors is required to generate and maintain T_RM_ cells in tissues (55). Notch and Runx3 have been reported to play an important role in T_RM_ cell differentiation in tumor microenvironments in both humans and mice, and in infectious contexts (35, 56). We have previously reported that the NFAT and SMAD signaling pathways triggered by the concomitant engagement of TCR and TGFBR, cooperate to induce CD103 expression in tumor-specific T lymphocytes and in the formation of CD8^+^ T_RM_ cells in a TGF-β-rich tumor microenvironment (43, 44, 51). Human NSCLC CD8^+^ T_RM_ cells display a particular molecular signature, with the expression of transcription factors specific to resident T cells, including NF-κB, BATF (basic leucine zipper transcription factor), Aiolos and AHR (aryl hydrocarbon receptor), which are probably involved in the maintenance and effector functions of these lymphocytes (17, 18). T-bet downregulation is required for the formation of memory T cells in mouse models, but residual T-bet is required to promote IL-15R signaling, thereby favoring the long-term survival of T_RM_ cells in peripheral tissues (57).

### 2.2 CD4^+^ T_RM_ cells

The identification of human CD4^+^ T_RM_ cells has remained challenging, partly due to the presence of functionally different helper T-cell (T_H_) subpopulations and their plasticity, making it difficult to identify a common CD4^+^ T-cell memory precursor (58, 59). Nevertheless, CD4^+^ T_RM_ cells have been characterized in both mouse and human lymph nodes, salivary glands, skin, gut, spleen, brain and lungs, mostly in infectious contexts (60–65). Human CD4^+^ T_RM_ cells have several transcriptional and phenotypic features in common with CD8^+^ T_RM_. Indeed, CD4^+^ T_RM_ cells express the CD69 lectin and have an effector-cell memory phenotype, with a CD45 RA^-^ CCR7^-^ and CD62L^-^ profile at diverse anatomic sites, including the spleen, lymph nodes, lungs, tonsils, salivary glands and intestine (61, 64, 66, 67).

Experiments in various infectious models have shown that CD4^+^ T_RM_ cells play a protective role. In this context, the adoptive transfer of lung CD4^+^ T_RM_ cells provides better protection against influenza infection than CD4^+^ circulating memory T cells (68, 69). In *Mycobacterium tuberculosis* infection, CD4^+^ T_RM_ cells migrate to the lung parenchyma more strongly than circulating memory T-cells, resulting in a greater reduction of bacterial load (70). In *Leishmania major* infections, CD4^+^ T_RM_ cells also play an important role in improving the effector functions of innate and adaptive immune cells and recruiting these cell populations to infected sites (71).

#### 2.2.1 Phenotype of CD4^+^ T_RM_ cells

The phenotypic and functional characterization of CD4^+^ T_RM_ cells was first described in infectious diseases. CD103 expression on CD4^+^ T cells differs between tissues, and CD103^+^CD4^+^ T_RM_ cells are mostly found in the skin, gut and lungs (61). Human dermis contains CD4^+^ memory T cells that do not express CD103, but the CD4^+^ memory T cells in the epidermis are CD103^+^ (63). Other key molecules involved in the tissue residency of CD4^+^ T cells include the CD49a integrin, which is present on CD4^+^ T_RM_ cells in normal brain tissues and lung tumors, and the CXCR6 chemokine receptor (61, 66, 67, 72). CD4^+^ T_RM_ cells also strongly express the CXCR3 and CCR5 chemokine receptors, which promote T-cell recruitment to inflamed tissues (73, 74). During influenza virus infection, CCR5 accelerates the recruitment of memory CD8^+^ T cells to the lung airways, and CCR5 deficiency in infected mice results in a decrease in T_RM_ cell recruitment and, thus, impaired control over viral replication during subsequent rechallenge (75). CD4^+^ T_RM_ cells display heterogeneous phenotypes according to their tissue residency, but additional markers of this T_RM_-cell subset need to be identified.

CD103^+^CD4^+^ T lymphocytes infiltrating human lung tumors express multiple inhibitory receptors, such as PD-1, CTLA-4 and 2B4 (CD244), suggesting that they are chronically stimulated and potentially exhausted (72). However, upon stimulation with anti-CD3 and anti-CD28 antibodies, these cells are able to secrete IFN-γ, TNF-α and IL-2 more efficiently than other TIL populations. CD103^+^CD4^+^ TIL also display increases in the levels of transcripts encoding inflammatory chemokines, such as CCL3, CCL4 and CCL5, and cytotoxic molecules, including IFNγ, granzyme A and granzyme B. These data suggest that the response to antigenic stimulation is rapid, despite the strong expression of inhibitory receptors (72).

#### 2.2.2 Cytokines and transcription factors involved in CD4^+^ T_RM_ cell formation and maintenance

Following the transfer of antigen-primed T cells into influenza-infected mice, IL-2 neutralization greatly decreases CD4^+^ T_RM_ cell formation, and a residual memory population is maintained independently of IL-2 signaling (68). Blockade with anti-IL-15 receptor antibodies results in a defect of memory cell recovery after infection, with no impact on the accumulation of effector T cells in the lungs, suggesting that the maintenance of CD4^+^ T_RM_ cells requires IL-15 (68). Moreover, the administration of anti-TGF-β neutralizing antibodies has no effect on the generation of CD4^+^ T_RM_ cells in the lungs. Overall, these studies suggest that the mechanisms by which CD4^+^ T_RM_ cells differentiate are different from those of CD8^+^ T_RM_ cells, particularly in terms of the need for TGF-β (68).

Transcriptional analyses investigating the transcription factors involved in CD4^+^ T_RM_ cell differentiation revealed an upregulation of the *ERG2*, *EPAS1*, *BATF* and *IRF4* genes in lung tumor CD4^+^ T_RM_ cells (66, 72). Increases in *PRDM1* (encoding the transcription factor Blimp-1) and *ZNF683* (encoding Hobit) transcript levels were also observed in CD4^+^ T_RM_ cells from lung tumors, with no increase in the level of Hobit protein (66, 72). The genes encoding the proteins of the Notch pathway are also upregulated in CD4^+^ T_RM_ cells, with transcript levels highest for *NOCTH1*, *RBPJ* and *JAG2* (66). However, the roles of these transcription factors in CD4^+^ T_RM_ cell formation and maintenance remain to be elucidated. Further studies are required to improve our understanding of the regulatory pathway and mechanisms of differentiation of CD4^+^ T_RM_ cells, particularly in tumor settings.

## 3 TGF-β, a master regulator for CD8^+^ T_RM_ cell differentiation

It is now widely accepted that TGF-β is required for the differentiation of CD8^+^ T_RM_ cells in infectious diseases and cancers (8, 35, 76–78). The TGF-β-dependent differentiation into CD8^+^ T_RM_ cells of CD8^+^ T lymphocytes from various tissues, including those of the intestine, skin and lungs, has been described (8, 79, 80). This process involves a decrease in the levels of Eomes and Tbet, which is essential for the differentiation of T_RM_ cells (57). TGF-β is known for its immunosuppressive effects and its role in T-cell exclusion from the tumor microenvironment. However, TGF-β is also a key regulator of CD103 expression in TCR-engaged CD8^+^ T cells and is involved in the activation of this integrin in CD8^+^ T_RM_ cells (41, 43, 44). Indeed, CD103 is induced in tumor-reactive T lymphocytes following TCR engagement with specific peptide-major histocompatibility complex (MHC)-class I complexes on cancer cells in a tumor microenvironment containing mature TGF-β. TGF-β is also involved in CD103 activation through the induction of integrin-linked kinase (ILK) phosphorylation. This leads to AKT phosphorylation and the initiation of integrin inside-out signaling, resulting in an increase in the affinity of CD103 for its ligand, E-cadherin (81). The purinergic receptor P2RX7 is required for T_RM_ cell sensitivity to TGF-β, and defects of P2RX7 in T cells result in a transient decrease in the expression of TGFBR2 and CD103, and weaker TGF-β signaling (77). The treatment of activated CD8^+^ T cells with a pharmacological agonist of P2RX7 leads to increases in *Itgae* and *Tgfbr2* mRNA levels, suggesting that P2RX7 signaling improves the response of CD8^+^ T cells to TGF-β, thereby promoting CD8^+^ T_RM_ cell differentiation (77).

**Figure 1:**
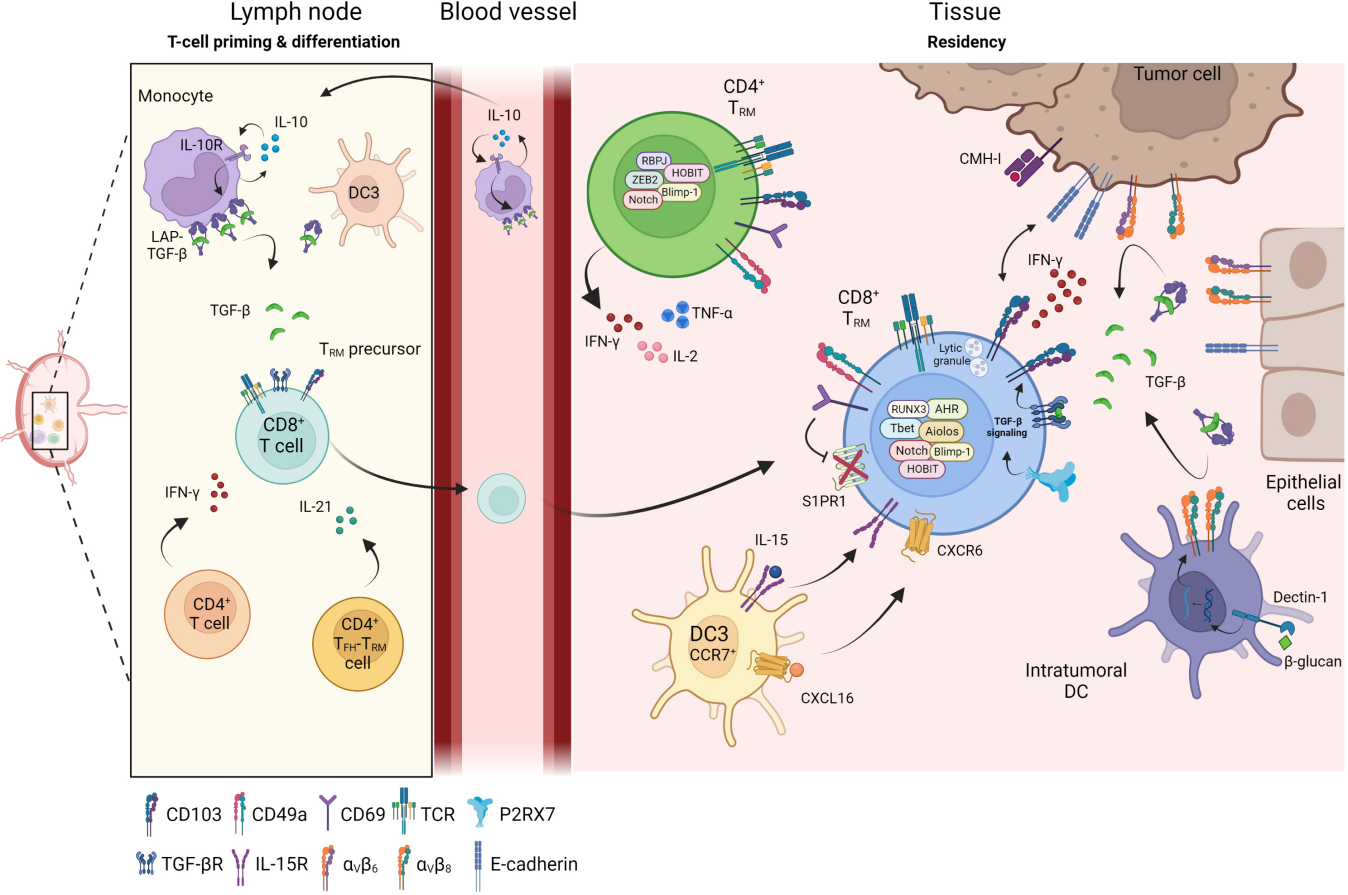
Differentiation of T_RM_ in tissue. Priming and differentiation of naïve CD8^+^ T cells take place in the lymph node in contact with monocytes or dendritic cells (DC3) which promote the generation of CD8^+^ T_RM_ precursors. Through cytokines secretion such as IFN-γ or IL-21, CD4^+^ T cells help CD8^+^ T cells to be fully functional and influence the T_RM_ differentiation. Primed CD8^+^ T_RM_ precursor cells reach the inflamed sites where they differentiate into CD8^+^ T_RM_. Within the tissue, several cellular components regulate and maintain T_RM_ cells: DC3 participate in positioning CD8^+^ T_RM_ cells in an immune niche and promote their survival, DC infiltrating tumors, tumor cells or epithelial cells express integrin α_v_β_6_ and α_v_β_8_ and transform LAP-TGF-β to active TGF-β. Active TGF-β maintains tissue residence of T_RM_ cells and initiates CD103 signaling leading to functional and cytotoxic T_RM_ cells. CD4^+^ T_RM_ cells have recently been identified and are also present in peripheral tissues and solid tumors where they secrete inflammatory cytokines such as IFN-γ, TNF-α or IL-2.

TGF-β is secreted in a latent, inactive form bound to latency-associated protein (LAP) and its activation is mediated by the cleavage of LAP by metalloproteinases and α_v_β_6_ and α_v_β_8_ integrins. These integrins are expressed by keratinocytes, and defects of α_v_β_6_ or α_v_β_8_ decrease the amount of mature TGF-β, in turn leading to a decrease in CD103 expression and T_RM_ density in the tissue (82). The specific inhibition of α_v_β_6_ integrin after T_RM_ establishment significantly decreases the number of these cells in epithelial tissues, with no consequences for their frequency in lymphoid organs and blood, providing further support for the conclusion that TGF-β is essential for the development and maintenance of T_RM_ cells (82). Cancer cells also express α_v_ integrins. They are therefore able to activate TGF-β and, thus, to promote *in situ* CD8^+^ T_RM_ cell differentiation and CD103 induction in CD8 T cells (83). In a mouse model based on the tamoxifen-inducible ablation of *Tgfbr2* in CD8^+^ T cells, only small numbers of CD8^+^ T_RM_ cells were detected in the skin, demonstrating the dependence of epidermal T_RM_ cells on an autocrine source of TGF-β (78). Moreover, virus-specific T_RM_ cells are more likely to persist in the epidermis than bystander T_RM_ cells when small amounts of active TGF-β are present in the skin (78). Overall, the differentiation of T_RM_ cells is orchestrated by antigen-dependent TCR activation and the Smad signaling pathway mediated by TGF-β, which is also required for the long-term maintenance of T_RM_ cells in tissues (**Figure 1**).

## 4 The interactome of T_RM_ cells

### 4.1 Interactome with tumor cells

In NSCLC, CD103^+^CD8^+^ T_RM_ cells migrate toward epithelial tumor regions more rapidly than effector T cells, which are retained in the stroma (16, 17, 25, 81). Most of the CD103^+^ cells in the tumor microenvironment are CD8 T cells, whereas most of the CD8^+^ TIL located in the stroma do not express this integrin (16, 25). This capacity of T_RM_ cells to migrate to the tumor islets is mediated by CD103 and enhanced by the presence of TGF-β (81). An accumulation of CD103^+^ T_RM_ cells in tumor epithelial regions has also been observed in ovarian and urothelial cell carcinomas and in endometrial adenocarcinoma (15, 21, 84).

The recognition by the TCR of specific antigenic peptides presented by MHC-class I complexes on tumor cells is the first step in the engagement of a target-directed T-cell response. Following this first signal, a second signal is required for the full activation of CD8 T cells and to trigger cytotoxic functions. In CD8^+^ T_RM_ cells, CD103 acts as a costimulatory molecule that binds to E-cadherin, initiating an outside-in signal that promotes T-cell effector functions (43, 45). Moreover, CD103 is a key molecule for triggering T_RM_ cell cytotoxic activity following interaction with E-cadherin on target cells (41). CD103 is recruited to the immunological synapse formed between T cells and autologous tumor cells, where it promotes polarization and the exocytosis of cytotoxic granules, leading to the lysis of target cells (42) (**Figure 1**). Tumor CD8^+^ T_RM_ cells are enriched in antigen-specific T cells, which are activated upon coculture with autologous tumor cells and can kill tumor targets (16, 17, 29). Cytotoxic activity toward malignant cells is mediated by the TCR and dependent on CD103 expression, as anti-CD103 neutralizing antibodies block CD103-E-cadherin interaction and inhibit target cell lysis (16, 17, 43). Remarkably, the loss of E-cadherin during epithelial-to-mesenchymal transition corresponds to a mechanism used by cancer stem-cell (CSC)-like cells to evade CD103^+^CD8^+^ T_RM_ cell-mediated recognition and destruction (85).

### 4.2 Interactome with dendritic cells

Dendritic cells (DC) are important regulators of the immune response through their interaction with T lymphocytes and their orchestration of T-cell activation via the presentation of antigenic peptides and production of specific cytokines and chemokines. In lung tissues, CD103^+^ DC trigger the upregulation of CD103 on activated CD8^+^ T cells in a TGF-β-dependent manner (76). In a virus model, DNGR-1^+^ (Clec9a) DC-mediated cross-presentation is essential for the optimal priming of T_RM_ cells through the production of cytokines such as IL-12 and IL-15 (86). Furthermore, mice in which α_v_ integrin expression by DC is defective, have lower levels of CD8^+^CD103^+^CD69^+^ T cells in the epidermis (87). Migratory DC expressing α_V_β_8_ have also been shown to be involved in T_RM_ cell formation by supplying TGF-β to naïve CD8^+^ T cells in the lymph nodes (87). A population of circulating CD88^-^CD1c^+^CD163^+^ DC (called DC3), the precursors of inflammatory DC, was recently reported to drive the activation of naïve T cells and their differentiation into CD103^+^CD8^+^ T cells expressing T_RM_ cell markers, such as the *NUSAP1, DUSP4, CXCR6* and *FASLG* genes, in a TGF-β-dependent manner (88). In the context of HIV vaccination, IL-10-secreting monocytes potentiate TGF-β production, leading to the generation of CD8^+^ T_RM_ cell precursors in non-human primates and mice (89).

A similar DC3-induced CD103^+^ T-cell gene signature has been identified in breast and lung cancers (19, 52, 61). In breast cancer, the intratumoral administration of the β-glucan curdlan, a ligand of dectin-1, reprograms tumor-infiltrating DC, favoring the generation of Th1 cells with a higher proportion of CD8^+^CD103^+^ T cells in tumors; this effect is abolished by the neutralization of TGF-β or inhibition of α_v_β_8_ integrin (90). CXCR6 regulates the accumulation and persistence of effector CD8^+^ T cells by supporting their survival in the tumor tissue (91). This chemokine receptor can position CD8^+^ cytotoxic T lymphocytes (CTL) in perivascular niches in the tumor stroma, which is enriched in DC3 (CCR7^+^ DC) expressing the ligand of CXCR6, CXCL16. DC3 also trans-present IL-15 to tumor-resident CD8^+^ T cells to maintain their survival in intratumoral areas (91). In a mouse melanoma model, CXCR6 expression has been shown to result in the presence of tumor-specific CD8^+^ T_RM_ cells within CXCL16^+^ DC clusters in the skin, ensuring the persistence and functioning of these cells (92). Several types of cells, including epithelial cells (such as keratinocytes), lung tumor cells (82, 93), and immune cells, such as DC (91, 92) and T cells (66), can secrete or present CXCL16 at their membrane. In head-and-neck tumors, intranasal immunization induces CXCL16 production in broncho-alveolar lavage (BAL) and pulmonary parenchyma, and is associated with the formation of tumor-specific T_RM_ cells (93). CXCR6 deficiency in T cells decreases the efficacy of anti-cancer vaccines and recruitment of CD8^+^ T_RM_ cells to tumors (50). The CXCR6-CXCL16 axis has also been shown to be crucial for the recruitment and maintenance of T_RM_ cells in the airways (50). DC and tumor cells play important roles in T_RM_ cell differentiation (**Figure 1**), but additional cellular and soluble factors promoting the formation, survival and localization of T_RM_ remain to be identified.

### 4.3 Interactome with CD4^+^ T cells

CD4^+^ T cells play an important role in CD8^+^ T-cell priming through the licensing of DC and the generation of a chemokine gradient within the draining lymph node. In infectious diseases, CD4^+^ T cells are required for the formation and function of memory T cells, and to confer cytotoxic and migratory capacities in T cells (94, 95). In the influenza infection model, CD4 T-cell depletion has no impact on the expansion and migration of memory CD8^+^ T cells into the lung parenchyma, but it does impair the expression of CD103 and CD69 on these cells in the tissue (96). Un-helped memory CD8^+^ T cells with low levels of CD103 expression are associated with changes in the ability of T cells to localize to the airway epithelium. By contrast, CD8^+^ T cells with CD4 T-cell help are associated with the recruitment of larger numbers of virus-specific CD8^+^ T cells and the expression of granzyme B upon heterosubtypic rechallenge (96). CD4^+^ T cells control CD103^+^CD8^+^ T_RM_ cell development and localization in the airway through IFN-γ signaling, and they also regulate the degree of exposure of CD8^+^ T cells to chemokines and cytokines, such as TGF-β, which regulate the expression of CD103.

The specific depletion of lung CD4^+^ follicular helper (T_FH_) T_RM_-like cells, which strongly express T_FH_-associated genes, including *Il21*, *Tox2*, and *Pdcd1*, results in a decrease in influenza virus-specific CD8^+^ T_RM_ cells (97). Late depletion, at the memory stage, of CD4^+^ T_RM_ cells decreases CD8^+^ T_RM_ cell responses, suggesting that lung CD4^+^ T_RM_ cell continuous help is required for the maintenance of CD8^+^ T_RM_ cells. This help is mediated by IL-21, as the administration of anti-αIL-21R antibodies after infection decreases antigen-specific CD8^+^ T_RM_ responses. Overall, these studies support the involvement of CD4^+^ T cells, and more specifically of CD4^+^ T_RM_ cells, in the development of CD8^+^ T_RM_ cells during infectious diseases. However, the help that these cells provide for tumoral CD8^+^ T_RM_ cell formation and function requires further investigation.

## 5 Functions of T_RM_ cells and their role in antitumor immune responses

T_RM_ cells are highly heterogeneous, differing considerably between tissues and diseases, and either within tissues (98, 99). There is a tremendous diversity in the levels of CD69, CD49a and CD103 expression observed, which can affect T_RM_ cell functions, including proliferation and the production of cytokines, such as TGF-β, type 1 interferon (IFN) and IL-12 (100–102). In lung tumors and the skin, a proportion of CD8^+^ T_RM_ cells displaying strong CD49a and CD103 expression can produce Th1 cytokines, such as IFN-γ and TNF-α, and IL-17, suggesting a particular function in the control of tumor progression (17, 103). The binding of CD49a to collagen IV has been reported to increase IFN-γ and TNF-α production (104). The capacity of T_RM_ cells to secrete IFN-γ and TNF-α provides evidence for a key role of these cells in antitumor immune responses and in the control of tumor progression in melanoma (105). In B16-Ova-engrafted mice, the blockade of VLA-1 (CD49a/CD29) or CD103 alters tumor growth control (38). CD49a and CD103 are important integrins for T_RM_ cell activation, migration and cytotoxic function within tumors.

Tumor CD8^+^ T_RM_ cells express a range of inhibitory receptors, such as PD-1, CTLA-4, and TIM-3, together with co-activation molecules, such as 4-1BB (16–19). The co-expression of PD-1 and 4-1BB by CD8^+^ TIL in lung tumors is associated with a higher density of TIL (18). Despite the expression of these inhibitory receptors, which suggests an overlapping phenotype with exhausted T cells, T_RM_ cells upregulate TCR activation genes (*NR4A1, TNFRSF9 [4-1BB], CD69*), secrete more TNF-α and IFN-γ, and are more cytotoxic than other CD8^+^ TIL (16–19, 26). This gene signature is associated with higher levels of proliferation and inflammatory cytokine production by CD8^+^ T_RM_ in early-stage NSCLC (106). In head-and-neck (29) and lung (17) cancers, CD8^+^CD103^+^ TIL also strongly express the CD39 ectonuclease. CD8^+^ TIL co-expressing CD103 and CD39 are tumor-reactive and have a resident memory profile (18, 29, 107), with a restricted TCR repertoire (17, 18, 29). CD103^+^CD8^+^ TIL produce large amounts of cytotoxic molecules, such as granzyme B, and can kill autologous tumor cells (16, 17, 19, 26). These T_RM_ cell features may account for the better overall survival (OS) observed in patients with tumors displaying high levels of CD8^+^CD103^+^ TIL infiltration (**Table 1**). In tumors, T_RM_ cells and exhausted T cells display similar phenotypes and anti-tumor effector functions following anti-PD-1 treatment (16, 17, 26). However, transcriptional and functional studies of tumor T_RM_ cells revealed specific differentiation program and molecular features distinct from non-T_RM_ cells (17).

**Table 1:** Prognostic value of CD103^+^CD8^+^ T_RM_ cells in various types of cancer.

Tumor type	Observations	References
Bladder cancer	High CD103^+^ TIL densities are a good prognostic factor (DFS and OS)	(21)
Breast cancer	High CD8^+^CD103^+^ intratumoral T cell densities are a good prognostic factor in basal-like subsets (OS)	(108)
	The CD8^+^ T_RM_ cell signature is associated with a good prognosis (DFS and OS)	(19)
	An enrichment in CD8^+^CD103^+^ T_RM_ cells in tumoral islets is associated with a good prognosis (relapse-free)	(109)
Cervical cancer	CD103 expression is a good prognostic factor for disease-specific survival	(110)
Colorectal cancer	CD8^+^CD103^+^ T cells are more important in the tumoral epithelium than in healthy epithelium	(20)
Endometrial cancer	High CD8^+^CD103^+^ T-cell densities are a good prognostic factor (disease-specific survival)	(84)
Esophageal cancer	High CD8^+^CD103^+^ T_RM_ cell densities are a good prognostic factor (OS)	(111)
Gastric cancer	CD8^+^CD69^+^CD103^+^ T_RM_ cell densities are a good prognostic factor (OS) but these cells are less frequently present in metastatic cancers	(112)
	High intratumoral CD8+CD103^+^ T cell densities are associated with better OS	(113)
	CD8^+^CD103^+^ T_RM_ cell numbers are a good prognostic factor (OS and DFS)	(114)
Head-and-neck cancer	CD8^+^CD103^+^CD39^+^ TIL densities are a good prognostic factor (OS)	(29)
Hepatocellular carcinoma	High CD103^+^PD-1^+^ T-cell numbers are associated with good prognostic factor (DFS)	(115)
	HBV-specific CD8^+^ T cells numbers defined as expressing CD69^+^ and CD103^+^ are good prognostic factor (Tumor relapse-free survival)	(116)
Lung cancer	An enrichment in CD8^+^CD103^+^ TIL in the epithelium is associated with a good prognosis (OS)	(16)
	CD8^+^CD103^+^ TIL densities are a good prognostic factor (OS)	(18)
	CD8^+^CD103^+^ TIL densities are a good prognostic factor (OS)	(25)
	CD8^+^CD103^+^ TIL densities are a good prognostic factor (OS)	(17)
Melanoma	CD8^+^VLA-1^+^ TIL are associated with better survival (OS and DFS)	(38)
	CD8^+^ CD103^+^ T_RM_ cell densities are a good prognostic factor (survival proportion)	(117)
Oropharyngeal cancer	High levels of CD8^+^CD103^+^ T_RM_ are associated with a good prognosis (OS)	(118)
Ovarian cancer	CD8^+^CD103^+^ TIL are more frequent in high-grade cancers than in other cancers and are a good prognostic factor (DFS)	(15)
	CD8^+^PD-1^+^CD103^+^ TIL densities are a good prognostic factor (OS)	(24)
	CD3^high^CD103^high^ TIL densities are a good prognostic factor (OS)	(119)
Pancreatic cancer	The ratio of intraepithelial TIL levels to CD8^+^CD103^+^ TIL levels is a good prognostic factor (DFS and OS)	(120)

Non-exhaustive. TIL, tumor-infiltrating lymphocytes; OS, overall survival; DFS, disease-free survival.

## 6 Relevance of T_RM_ cells for cancer immunotherapy

### 6.1 T_RM_ cells as a biomarker of the response to therapeutic cancer vaccines

The role of T_RM_ cells in immune responses to tumors makes them particularly relevant as potential biomarkers of responses to cancer immunotherapies. In melanoma patients treated with the Melan-A peptide vaccine, circulating VLA-1-expressing Melan-A-specific CD8^+^ T cell numbers are correlated with better OS (38). Intranasal STxB-E7 vaccination has a protective effect in a mouse orthotopic head-and-neck tumor model, and this effect is suppressed by the *in vivo* blockade of CD49a, through decreases in CD8^+^ TIL densities (39). Moreover, the *in vivo* blockade of TGF-β results in decreases in the number of tumoral CD8^+^ T_RM_ cells and the benefits of vaccination (25). In a similar model, the combination of an intramuscular vaccine targeting HPV-E6 and -E7 with irradiation triggered the formation of larger numbers of CD103^+^ cells in TC1 tumors than either of these therapies used alone (110). In the mouse B16F10-Ova melanoma model, intradermal vaccination induces a stronger accumulation of CD8^+^CD69^+^CD103^+^ T_RM_ cells than intraperitoneal immunization and results in stronger protective effects due to the local protective effect of T_RM_ cells (121). Local mucosal vaccination therefore promotes the formation of T_RM_ cells, which can be used as a biomarker of the response to the cancer vaccine and of an optimal immune response.

### 6.2 T_RM_ cells as a biomarker of the response to immune checkpoint inhibitors

Inhibitory receptors have been reported to inhibit the effector functions of T_RM_ cells during long-term exposure to the antigen (122, 123). Tumor T_RM_ cells express a wide range of inhibitory receptors (16–18, 35, 124). Immune checkpoint inhibitors (ICI) would therefore be expected to restore, or boost the functional activities of T_RM_ cells. In NSCLC and hepatocellular carcinoma (HCC), the inhibition of PD-1 on tumor CD103^+^CD8^+^ T cells stimulated with anti-CD3 plus anti-CD28 antibodies enhances IFN-γ production by these cells (115, 125) (**Table 2**). Blockade of the PD-1-PD-L1 interaction also increases the cytotoxicity of CD8^+^ T_RM_ cells toward tumor cells (16). In a cohort of NSCLC patients treated with anti-PD-(L)-1, tumors with high levels of CD103^+^CD8^+^ TIL had the best responses to immunotherapy (17) (**Table 2**). The use of T_RM_ cells as a predictive biomarker of the response to immunotherapies has been also documented in urothelial cancer and NSCLC patients harboring tumors with high levels of *ITGAE* transcripts treated with anti-PD-L1 (126). This finding supports the conclusion that CD8^+^ T_RM_ cells can be used as a biomarker of the response to ICI. In addition, low levels of α_v_ integrin in tumor cells are associated with higher levels of T_RM_ cell infiltration into lung tumors and a better response to anti-PD-(L)1 (83).

**Table 2:** Effect of anti-PD-(L)1 immunotherapy on CD8^+^CD103^+^ T_RM_ cell and prognostic value.

Treatment	Tumor type	Effects	References
Anti-PD-1	Hepatocellular carcinoma	CD8^+^CD103^+^ T cells are increased in patient with stable disease compared with those with progressive disease	(115)
Anti-PD-1	Melanoma	CD8^+^ CD103^+^ T_RM_ cells are associated with a better prognosis than total CD8^+^ TILs and could initiate the response to anti-PD-1	(117)
Anti-PD-1	NSCLC	An increase in the density of CD8^+^CD103^+^ T_RM_ cells is associated with a response to ICI	(17)
Anti-PD-L1	NSCLC and urothelial carcinoma	Higher levels of *Itgae* transcripts are associated with a better response to ICI	(126)
An-i-PD-1	Esophageal cancer	CD8^+^CD103^+^ cells have stronger cytotoxic activity after anti-PD-1 treatment and are not affected by chemotherapy	(111)
Anti-PD-L1	Stomach cancer	Anti-PD-L1 treatment promotes the survival of CD8^+^CD69^+^CD103^+^ T_RM_ cells, which are associated with a response to treatment	(112)
Adoptive cell transfer and anti-PD-1	Melanoma	Anti-PD-1 treatment increases the infiltration of transferred T_CM_, which can give rise to T_RM_ cells	(127)

Non-exhaustive.

Remarkably, for patients treated with immunotherapy classified as “responders”, the administration of ICI increases the infiltration of CD103^+^CD8^+^ T cells into the tumor in NSCLC and melanoma (17, 117). This finding suggests that PD-1 blockade has an impact on T_RM_ cells, but it remains unclear if it directly affects intratumoral T_RM_ cells or has a more indirect effect on the newly recruited T cells that differentiate into T_RM_ cells within the tumor. In a PDX model of gastric adenocarcinoma, anti-PD-L1 therapy results in an increase in the proportion of T_RM_ cells among total CD8^+^ T cells (112). In an esophageal squamous cell carcinoma mouse model, PD-1 blockade induces an increase in the size of both the CD8^+^ and CD103^+^ cell populations (111). The proliferation and differentiation of stem-like TCF1^+^PD-1^med^ TIL are also important mechanisms involved in the response to ICI and therapeutic vaccination (128, 129). Deeper investigations of the role of T_RM_ cells in the response to cancer immunotherapies will help to improve both treatments and the management of patients.

## 7 Conclusion

The differentiation and maintenance of CD8^+^ T_RM_ cells across tissues are orchestrated by diverse cellular interactions, with immune cells, stromal cells or tumoral cells, and by soluble factors, including cytokines and chemokines, which are produced by these cells within the tumor microenvironment. In normal epithelial tissues and solid tumors, TGF-β is a key cytokine involved in the regulation of CD8^+^ T_RM_ cell formation and function. However, it remains unclear whether other signals are required for T_RM_ cell differentiation and persistence. Similar, many questions remain unanswered concerning the cytokines and signaling pathways regulating CD4^+^ T_RM_ cell development and maintenance in the tumor. CD8^+^ T_RM_ cells are emerging as a reliable biomarker of the response to cancer immunotherapies. However, even though T_RM_ cells are enriched in tumor-specific T cells endowed with cytotoxic functions, additional features of T_RM_ cells influencing the efficacy of cancer therapies remain to be characterized. Improvements in our understanding of the mechanisms involved in the differentiation and survival of T_RM_ cells within the tumor microenvironment would lead to the identification of promising novel targets for optimizing the efficacy of current cancer immunotherapies.

## Author contributions

ID, TT, FM-C and SC wrote, reviewed and/or revised the manuscript. All authors contributed to the article and approved the submitted version.
